# Modulation of miRNAs by Vitamin C in Human Bone Marrow Stromal Cells

**DOI:** 10.3390/nu10020186

**Published:** 2018-02-08

**Authors:** Ravindra Kolhe, Ashis K. Mondal, Chetan Pundkar, Sudharsan Periyasamy-Thandavan, Bharati Mendhe, Monte Hunter, Carlos M. Isales, William D. Hill, Mark W. Hamrick, Sadanand Fulzele

**Affiliations:** 1 Department of Pathology, Augusta University, Augusta, GA 30912, USA; rkolhe@augusta.edu (R.K.); amondal@augusta.edu (A.K.M.); cpundkar@augusta.edu (C.P.); 2 Cellular Biology and Anatomy, Augusta University, Augusta, GA 30912, USA; speriyasamy@augusta.edu (S.P.-T.); bmendhe@augusta.edu (B.M.); whill@augusta.edu (W.D.H.); mhamrick@augusta.edu (M.W.H.); 3 Department of Orthopaedic Surgery, Augusta University, Augusta, GA 30912, USA; mohunter@augusta.edu (M.H.); CISALES@augusta.edu (C.M.I.); 4 Institute of Regenerative and Reparative Medicine, Augusta University, Augusta, GA 30912, USA

**Keywords:** bone marrow stromal cells, vitamin C, miRNA

## Abstract

MicroRNAs (miRNAs) are small (18–25 nucleotides), noncoding RNAs that have been identified as potential regulators of bone marrow stromal cell (BMSC) proliferation, differentiation, and musculoskeletal development. Vitamin C is known to play a vital role in such types of biological processes through various different mechanisms by altering mRNA expression. We hypothesized that vitamin C mediates these biological processes partially through miRNA regulation. We performed global miRNA expression analysis on human BMSCs following vitamin C treatment using microarrays containing human precursor and mature miRNA probes. Bioinformatics analyses were performed on differentially expressed miRNAs to identify novel target genes and signaling pathways. Our bioinformatics analysis suggested that the miRNAs may regulate multiple stem cell-specific signaling pathways such as cell adhesion molecules (CAMs), fatty acid biosynthesis and hormone signaling pathways. Furthermore, our analysis predicted novel stem cell proliferation and differentiation gene targets. The findings of the present study demonstrate that vitamin C can have positive effects on BMSCs in part by regulating miRNA expression.

## 1. Introduction

Adult stem cell differentiation is a complex process that is heavily influenced by tissue origin and interaction with the cellular microenvironment [1]. Bone marrow stromal cells (BMSCs) are mesenchymal lineage cells that can differentiate into a number of different cell types including osteoblasts, osteocytes, adipocytes, and chondrocytes [2,3]. The differentiation pathway of BMSCs depends in large part on their niche/microenvironment [1]. Targeted gene reprogramming is vital to direct BMSCs toward specific lineages in the field of tissue-engineering. One potential strategy in this regard employs various micronutrient supplements such as amino acids and vitamins to guide BMSC differentiation.

Micronutrients, including vitamin C, are important factors in musculoskeletal development and BMSC biology [4,5,6,7,8,9,10,11,12,13,14,15]. For example, vitamin C is a key player in collagen synthesis, cell proliferation, and BMSC differentiation [9,10,11,12,13,14,15]. Moreover, vitamin C acts as an antioxidant and prevents oxidative damage of cellular macromolecules. Vitamin C is an essential nutrient that is not synthesized by most mammals including humans [16,17,18] because of a mutation in gulonolactone oxidase (GULO) enzyme. GULO is an important catalyzing enzyme which helps in the conversion of l-gulono-g-lactone into ascorbic acid [17,18]. Human clinical and animal studies have demonstrated that a deficiency of this vitamin leads to musculoskeletal deformities [19,20]. Therefore, dietary supplementation of vitamin C is essential for the normal function of BMSCs. Vitamin C stimulates BMSC differentiation by induction of differentiation-specific genes such as Collagen type 1, RUNX2, and ALP [11]. Furthermore, vitamin C is also known to regulate human embryonic stem cell differentiation through epigenetic regulation [21,22].

The role of vitamin C in mesenchymal and embryonic stem cell differentiation has been investigated extensively [21,22,23,24]. It is well-established that vitamin C can alter gene expression in embryonic stem cells through epigenetic regulation by directly regulating Tet activity and DNA methylation [19,20,22,23]. Epigenetic regulation is a mechanism in which there is change in gene regulation without change in genetic makeup [25]. Epigenetic factors such as DNA methylation and microRNAs are known for their roles in BMSC differentiation and musculoskeletal development [26,27,28,29,30,31]. Vitamin C and its role in DNA methylation have been well-studied in different biological systems [32,33,34,35,36,37] but not much is known with regard to miRNA and vitamin C. MicroRNAs are small, non-coding, endogenous, single stranded RNAs comprised of 22 nucleotides that bind to the 3′ untranslated region of target messenger RNA (mRNA) [38,39]. MicroRNA negatively regulates gene expression at the post-transcriptional level by degrading mRNA or by inhibiting translation [38,39]. It has been reported in a number of studies that miRNA regulates almost all cellular events including cell proliferation, differentiation, and development [40,41,42,43]. Vitamin C-dependent miRNA gene regulation has not been studied previously in human BMSCs. For this study, we collected human bone marrow and isolated bone marrow stromal cells. The osteogenic and adipogenic differentiation properties of the cells were analyzed. The BMSCs were treated with vitamin C followed by miRNA array. The selected miRNAs were further conformed using real time-polymerase chain reaction polymerase chain reaction (PCR). Bioinformatics analyses were performed on differentially expressed miRNAs to identify novel target genes and signaling pathways. Our data demonstrated that vitamin C regulates a number of miRNAs and plays an important role in various stem cell-signaling pathways.

## 2. Materials and Methods

### 2.1. Isolation of Human BMSCs (hBMSCs)

All work described here was approved by the Institutional Review Board and Institutional Biosafety Committee of Augusta University (AU). We collected bone marrow aspirates that were removed as part of orthopedic (total knee, hip, and ACL) surgeries and would normally have been discarded. Bone marrow was obtained under sterile conditions from orthopedic surgery patients (*n* = 10). The CD271 positive (+) BMSCs were isolated according to the manufacturer’s protocol using a kit (Miltenyi Biotec Inc., 130-092-283, Sunnyvale, CA, USA) following previously published methods [44,45]. The CD271+ MSCs were isolated directly from bone marrow, washed with standard culture medium composed of DMEM medium (Corning, 10-013-CM, NY, USA), 1% antibiotics-antimycotics (Invitrogen, 15240-062, Carlsbad, CA, USA) and 10% Fetal bovine serum (FBS), transferred to 100 mm culture dish and incubated at 37 °C in a humidified atmosphere at 5% carbon dioxide (CO_2_). The media with non-adherent cells was removed after 24 h, the adherent cells carefully washed in Phosphate-buffer saline (PBS), and adherent cells further expanded in fresh standard culture medium. Culture-expanded CD271 + BMSCs of passage 1 were used for treating with the vitamin C, miRNA array, quantitative real-time polymerase chain reaction (qPCR) and cell differentiation assay.

### 2.2. Osteogenic and Adipogenic Differentiation Assays

The differentiation potential of cultured hBMSCs into osteogenic and adipogenic lineages was validated in vitro. The osteogenic differentiation [12] and adipogenic assays [2] were performed as per published methods. In brief, cells were plated in 24-well plates at 5000 cells/cm^2^ and cultured in Dulbecco’s Modified Eagle Medium (DMEM) for 24 h. Culture medium was then aspirated and replaced with osteogenic medium. The osteogenic media was prepared in DMEM that was supplemented with 5% FBS, 0.25 mM ascorbic acid (Sigma-Aldrich, A4544, St. Louis, MO, USA), 0.1 mM dexamethasone (Sigma-Aldrich, D4902, St. Louis, MO, USA), and 10 mM *β*-glycerophosphate (Sigma-Aldrich, G9891, St. Louis, MO, USA). The medium was replaced freshly 2 times per week for 3 weeks. Osteogenic differentiation was assessed by staining for bone mineralization with Alizarin Red (AR; Sigma-Aldrich, A5533, St. Louis, MO, USA). The cells were fixed with 10% formalin for 20 min at room temperature (RT) and stained with 40 mM AR, pH 4.1 for 20 min at RT. Stained monolayers were visualized by phase-contrast microscopy using an inverted microscope (Nikon, Melville, NY, USA). Differentiation was quantified as previously described [2]. In brief, cells were destained using 10% cetylpyridinium chloride (Sigma-Aldrich, C0732, St. Louis, MO, USA). Collected samples were then analyzed with a microplate reader at 590 nm.

For the adipogenic assay, the cultures were incubated in IMDM (Gibco, 12440-046, Waltham, MA, USA) supplemented with 10% FBS, 10% Horse Serum (BioAbChem, 720460, Ladson, SC, USA), 12 mM l-glutamine, 5 μg/mL insulin (Cell Application Inc., 128-100, San Diego, CA, USA), 50 μM indomethacin (Sigma-Aldrich, I7378, MO, USA), 1 × 10^−6^ M dexamethasone, and 0.5 μM 3-isobutyl-1-methylxanthine (Sigma-Aldrich, I5879, St. Louis, MO, USA). The medium was replaced 2 times per week for 3 weeks followed by real time PCR on adipogenic genes.

### 2.3. Vitamin C Treatment, Gene Expression Analysis and Intracellular Vitamin C Estimation

Human BMSCs were cultured on 24 well plates and treated with or without vitamin C (low (25 µM) and high (100 µM) concentration) for 6 days. Media was changed every other day with or without vitamin C. Total RNA was isolated for gene expression analysis on both low and high dose vitamin C treatment groups. Collagen type II, BMP-2, BMP-7, RUNX-2 and OSX gene expressions were performed using real time PCR (Primers’ details in supplemental Table S1). Intracellular vitamin C estimation was performed using OxiSelect™ Ascorbic Acid Assay Kit (FRASC) (Catalog Number, STA-860, Cell Biolabs, Inc., San Diego, CA, USA). Briefly, hBMSCs were treated with (100 µM) and without vitamin C for 6 h followed by intracellular vitamin C estimation as per manufacturer’s protocol.

### 2.4. Microrna Array and Bioinformatics Analysis

The microRNA array was performed only on samples treated with the high dose (100 µM) of vitamin C. miRNAs were isolated using an miRNA isolation kit (SABiosciences Corporation, Frederick, MD, USA) that specifically captures small RNAs with length of less than 200 nucleotides as per the manufacturer’s protocol. RNA concentrations were determined using a NanoDrop 1000 Spectrophotometer (NanoDrop Technologies, Wilmington, DE, USA). The quality of RNA samples was characterized on an Agilent BioAnalyzer (Agilent Technologies, Santa Clara, CA, USA) using an RNA6000 Nano Chip (Agilent). Microarrays were performed on miRNA using an Affymetrix GeneChip^®^ miRNA 2.0 array at the Integrated Genomics Core, Augusta University, GA, USA. Details of the procedure can be found online at http://www.augusta.edu/cancer/research/shared/genomics. The miRNA profile was analyzed for hierarchical clustering of miRNA to generate heat maps. The results were normalized using robust multichip averages. *T*-tests were used to calculate the *p*-value to determine whether there is a significant difference for miRNA expression between the control and the treatment groups. Principal component analysis (PCA) was performed between vitamin C treatment and control samples. Gene Ontology (GO) and Kyoto Encyclopedia of Genes and Genomes (KEGG) signaling pathway analyses were performed using DIANA-miRPath v. 3.0 (http://diana.imis.athena-innovation.gr/DianaTools/index.php) on differentially expressed microRNAs target genes. GO word clouds were generated using the online Wordle software (www.wordle.net). Bioinformatics software (http://www.targetscan.org/vert_71/ and http://www.mirdb.org/) was used to predict targets genes of differentially regulated miRNAs of musculoskeletal importance.

Validation of miRNA using real time-PCR: Two hundred nanograms of enriched small RNA were converted into cDNA using RT2 miRNA First Strand Kit (SABiosciences Corporation, Frederick, MD, USA). Fifty picograms of cDNA were amplified in each qRT-PCR using syber green dye and miRNA specific primers. The real-time qRT-PCR was performed on a Bio-rad q-pcr machine with following cycling parameters: 95 °C for 10 min, then 40 cycles of 95 °C for 15 s, and 60 °C for 30 s. SYBR Green fluorescence was recorded during the annealing step of each cycle. The average of RNU6 (RNA, U6 small nuclear 2) and SNORD (small nucleolar RNA, C/D box) was used as normalization reference genes for miRNAs. Relative expression of miRNA was evaluated by using the comparative cycle threshold (CT) method (ΔΔ*C*t).

## 3. Results

### 3.1. Osteogenic and Adipogenic Differentiation Assays

**Figure 1:**
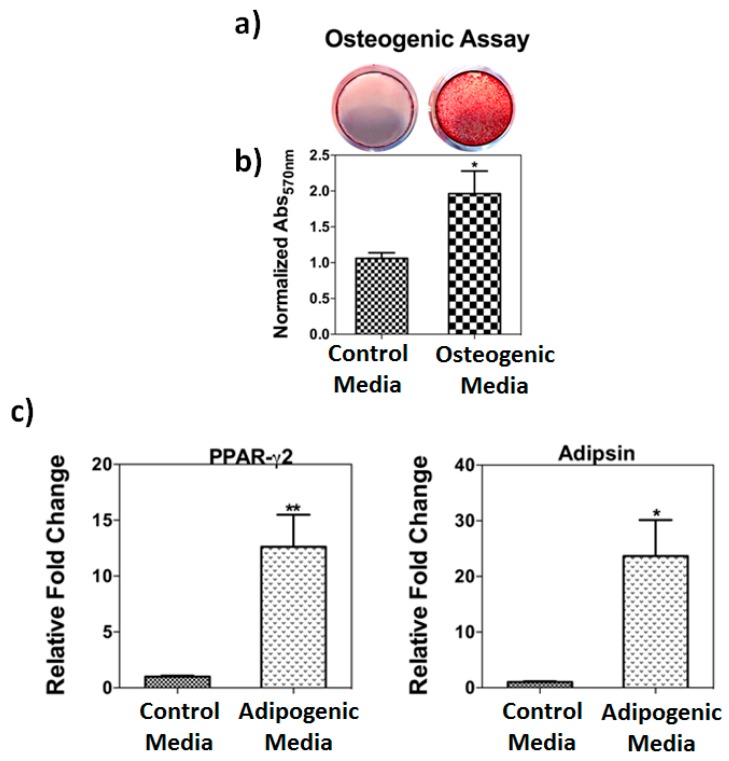
(**a**) Human bone marrow stromal cells (BMSCs) cultured in osteogenic medium and stained for mineralized nodules using an Alizarin red S assay; (**b**) Quantitative analysis of the extent of mineralization in the Alizarin red S assay using elution of dye by 10% (wt./vol.) cetylpyridinium chloride (means ± SD, *n* = 4) (**c**) Real time PCR analysis of steady-state levels of mRNA for adipogenic genes PPAR-g and adipsin. Data for each sample were normalized with glyceraldehyde-3-phosphate dehydrogenase (GAPDH) mRNA. Data (means ± SD, *n* = 4) are represented as the fold change in expression compared to the control. * *p* = 0.04, ** *p* = 0.01.

Human bone marrow stromal cells showed stem cell characteristics such as adhesion to tissue culture plates, fibroblast-like morphology, and differentiation properties. To identify their differentiation properties, hBMSC were cultured in osteogenic and adipogenic media. For osteogenic properties, cells were treated with osteogenic media for 3 weeks followed by Alizarin red assay staining and real time PCR. Treatment with osteogenic media showed significant (*p* = 0.01) increases Alizarin red staining (Figure 1) and bone specific markers (data not shown). hBMSCs were treated with adipogenic media for 3 weeks showed significantly (*p* = 0.01) elevated levels of adipogenic markers such as PPAR-g2 and Adipsin genes) (Figure 1c).

### 3.2. Vitamin C Regulates Musculoskeletal-Related Gene Expression in Hbmscs

**Figure 2:**
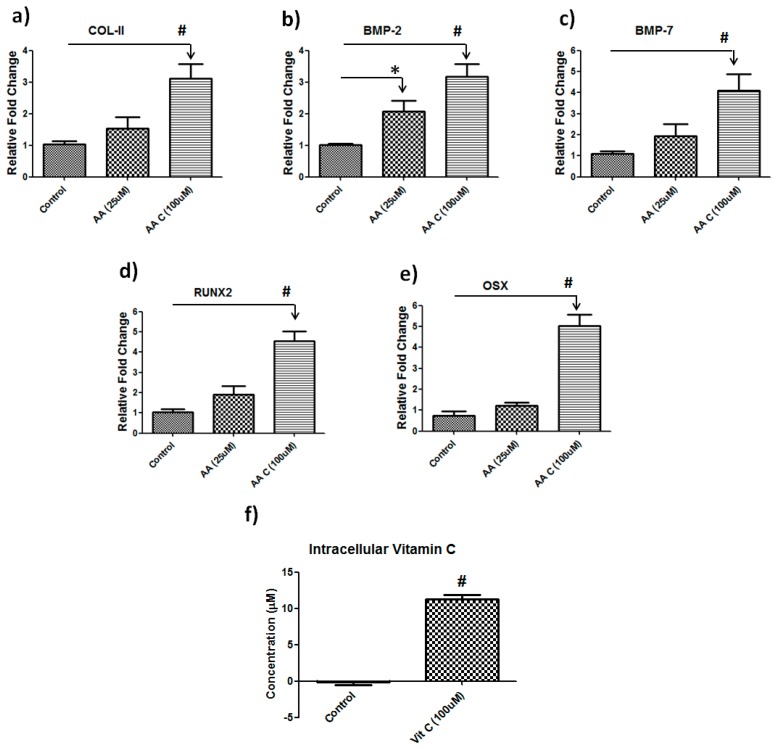
Vitamin C regulates musculoskeletal genes in hBMSCs. Real-time PCR showing dose dependent change in gene expression in hBMSCS after vitamin C treatment (**a**) Collagen II; (**b**) BMP-2; (**c**) BMP-7; (**d**) RUNX2 and (**e**) OSX (Osterix). Data (*n* = 4) are represented as the fold change in expression compared with control (* *p* = 0.04, # *p* = 0.001); (**f**) Intracellular concentration of vitamin C in hBMSCs. hBMSCs cells were treated with 100 μM vitamin C and intracellular accumulation of vitamin C was measured in the control and vitamin C treated cells after 6hrs (*n* = 6, # *p* = 0.001).

Supplementation of vitamin C is required for the differentiation of hBMSCs but the dose of vitamin C supplementation is debatable. To gain further insight into the dose of vitamin C in the human bone marrow stromal cell culture system, we treated cells with either a low (25 µM) or a high (100 µM) doses of vitamin C for 6 days followed by real time PCR on musculoskeletal related genes. A higher dose of vitamin C treatment showed the most significant changes in expression of those genes related to osteogenic differentiation. Specifically, COL-II and BMP2 were increased three-fold (*p* = 0.001), where BMP-7, RUNX2 and Osterix (OSX) were up-regulated approximately four-fold (*p* = 0.001) at a higher dose compared to control (Figure 2). Lower doses of vitamin C did not show significant changes (Figure 2). We also quantified intracellular accumulation of vitamin C following vitamin C treatment after 6hrs. We found significantly (*p* = 0.001) higher concentration of intracellular vitamin C compared to the control (Figure 2f).

### 3.3. Global Mirna Expression Profile Following Vitamin C

**Figure 3:**
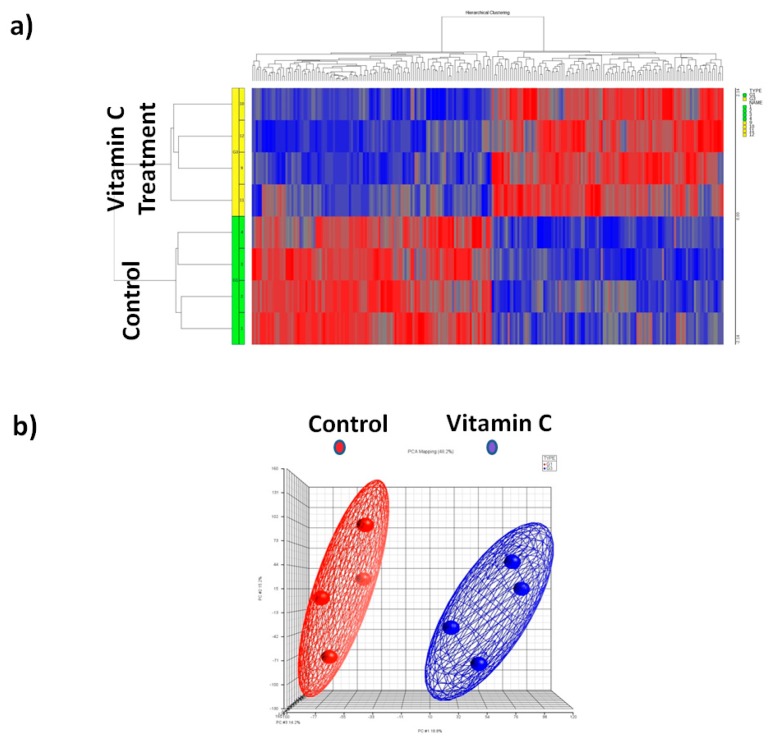
Differential miRNA expression in human bone marrow stromal cells after vitamin C treatment (*n* = 4 each group). (**a**) The heat-map showing the differential expression pattern of miRNAs compared to control group; (**b**) Principle component analysis (PCA) mapping of vitamin C treatment and control samples. Control group (indicated by red color) was clustered distinctly from vitamin C treated group (indicated by blue color).

To identify miRNAs that were differentially expressed following vitamin C treatment, we conducted a comprehensive miRNA microarray analysis of samples from hBMSCs that were treated with or without vitamin C. miRNAs were isolated after 6 days post treatment. The miRNAs that exhibited a significant (*p* < 0.05) 1.5-fold difference in expression after vitamin C treatment compared with the control groups were selected for analysis. Our analysis identified 122 miRNAs that were differentially expressed (*p* < 0.05) in response to vitamin C. Out of 122 miRNAs, 80 miRNAs were up-regulated and 42 were down-regulated in the presence of vitamin C. The list of up-regulated and down-regulated miRNAs after vitamin C treatment is shown in Table 1. Hierarchical cluster analysis using the normalized miRNA expression data confirmed that the expression of miRNAs in vitamin in C treated BMSCs can be clearly distinguished from the controls (Figure 3).

### 3.4. Principal Component Analysis (PCA)

We performed a PCA to explore the relationships between the control and vitamin C-treated samples. The PCA graph (Figure 3) shows the presence of the cluster of vitamin C treatment samples that is clearly distinct from the control (non-treated) samples.

### 3.5. Validation of Differentially Expressed Mirnas

**Figure 4:**
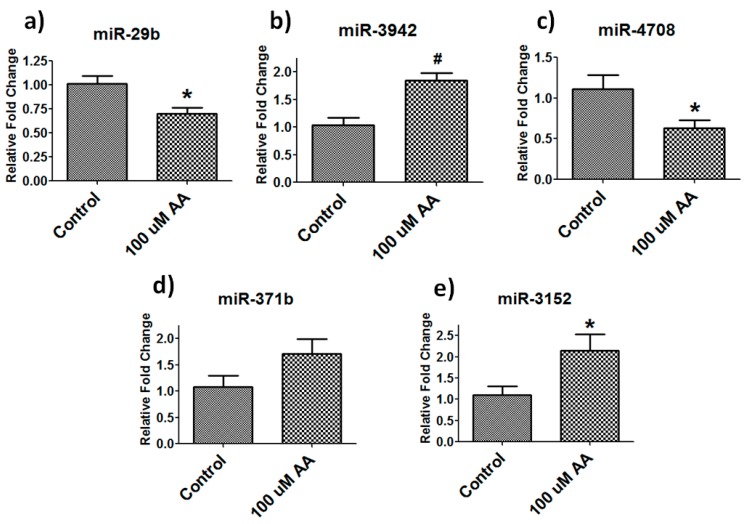
Validation of miRNA array data on randomly picked miRNAs. Real-time PCR showing change in miRNA expression in vitamin C (100 µM) treated samples compared to control (**a**) miR-29b; (**b**) miR-3942; (**c**) miR-4708; (**d**) miR-371b and (**e**) miR-3152 (*n* = 4–6, * *p* = 0.05, # *p* = 0.01).

To further verify the results obtained from miRNA microarrays, we performed real-time PCR on five randomly selected miRNAs to validate our findings. MiRNA real-time PCR showed similar changes as noted in miRNA array (Figure 4). In vitamin C (100 µM)- treated samples, we found that miR-29b (*p* = 0.04) and miR-4705 (*p* = 0.04) were significantly down-regulated and miR-3942 (*p* = 0.01) and miR-3152 (*p* = 0.04) were significantly up-regulated whereas miRNA-371b showed a trend of up-regulation similar to our array findings. Low dose vitamin C (25 µM) treatment did not show significant changes in any of the above-mentioned miRNA expression analyses (supplementary Figure S1).

### 3.6. Signaling Pathway Predictions

We performed Kyoto Encyclopedia of Genes and Genomes (KEGG) pathway annotation and GO analysis to identify functions of the miRNAs found to be differentially expressed after vitamin C treatment. The KEGG annotation analysis showed that a number of molecules are affected by these miRNAs. Interestingly, up-regulated and down-regulated miRNAs both regulate some common signaling pathways such as cell adhesion molecules (CAMs), fatty acid biosynthesis/metabolism and thyroid hormone signaling pathway. The important KEGG signaling for up-regulated miRNAs are Mucin type O-Glycan biosynthesis, glycosphingolipid biosynthesis, biotin metabolism and arrhythmogenic right ventricular cardiomyopathy (ARVC) and down-regulated are amino sugar and nucleotide sugar metabolism, endocytosis, MAPK signaling pathway, GABAergic synapse, and glutamatergic synapse. Details of KEGG annotation analysis are shown in Table 2.

The gene ontology analysis showed that more than 87 biological processes were associated with the down- and up-regulated miRNAs (Table 3). The most common GO pathways regulated by both up-regulated and down-regulated miRNAs are organelles, biosynthetic processes, cellular protein modification process, enzyme binding, cellular component assembly and nucleic acid binding transcription factor activity. Details of the GO analyses are shown in Table 3. Wordle-based clouds were generated for both up-regulated and down-regulated pathways from the GO analysis to identify most prominent vitamin C-dependent, miRNA-mediated signaling pathways (Figure S2). Word clouds demonstrate the font size depending on relative word frequencies in the GO analysis [46].

### 3.7. Bioinformatics Mirna Target Prediction

Based on miRNA targets predicted from the *in silico* analysis, we can derive some functional predictions of the differentially regulated miRNAs. We analyzed the potential targets of miRNAs that are differentially expressed following vitamin C, with the criteria that the miRNAs must bind the 3′-UTR of the mRNA with its seed sequence. We used Targetscan.org and mirdb.org target prediction tools to identify miRNA targets and their signaling pathways. We identified a number of miRNA targets of musculoskeletal and stem cell differentiation related genes. The lists of miRNA targets are shown in Table 4.

## 4. Discussion

Adult bone marrow stromal cells are an important cell type in regenerative, cell-based therapeutics [47]. Differentiation and regenerative capabilities of BMSCs can be enhanced by manipulating the cellular micro-environment. One goal of regenerative medicine is to optimize the regenerative potential of BMSCs through nutritional supplementation [4,5,6,7,8,9,10,11,12,13,14,15]. Vitamin C is one of the most important players in cell proliferation, differentiation, extracellular matrix (ECM) synthesis and cytoskeletal development [21,22,23,24]. We hypothesized that supplementation of vitamin C might regulate miRNA-dependent gene regulation. A number of reports have demonstrated that micronutrients such as vitamin E, D and amino acid derivatives regulate miRNA-dependent gene expression in various cell types [48,49,50,51,52]. Kim et al. (2015) recently demonstrated that dietary supplementation of a high dose of vitamin C resulted in enhanced anti-senescence and anti-atherosclerotic effects via regulation of anti-inflammatory microRNA [53]. Goa et al. (2014) reported that vitamin C induces a pluripotent state in mouse embryonic stem cells by modulating microRNA expression [54]. Another investigator also noted differential miRNA expression in vitamin C-deficient (l-gulonogammalactone oxidase knockout) C57BL6 mice during follicular maturation [55].

To our knowledge, no study has investigated the effect(s) of vitamin C-dependent miRNA regulation on human bone marrow stromal cells. Hence, identification of vitamin C-dependent microRNA regulation is important for understanding the basic mechanisms underlying BMSC differentiation and tissue engineering. We identified a list of miRNAs that were differentially expressed following vitamin C treatment (100 µM dose). Principal component analysis (PCA) showed that control samples are clustered together and clearly separated from vitamin C treatment groups. To further verify our miRNA array data, we randomly selected five miRNAs and performed real time PCR for further confirmation. Real-time PCR showed similar patterns of differential expression identical to those seen with the miRNA Array data.

Vitamin C is an essential nutrient that is not synthesized by most mammals including humans; therefore, the dietary supplementation of vitamin C is required for normal cell function. One outstanding question regarding vitamin C supplementation is what doses promote the optimal differentiation of human BMSCs. Our data demonstrate that a higher doses (100 µM) of vitamin C effectively activate musculoskeletal-related genes in human BMSCs whereas lower doses (25 µM) of vitamin C did not. Similar results were also noted for vitamin C-dependent miRNA regulation. At lower doses of vitamin C, we did not find a significant change in miRNA expression comparable to what we observed with the higher 100 µM dose. Our study suggested that dose of vitamin C (100 µM) is important to achieve miRNA and gene expression changes in human BMSCs.

**Figure 5:**
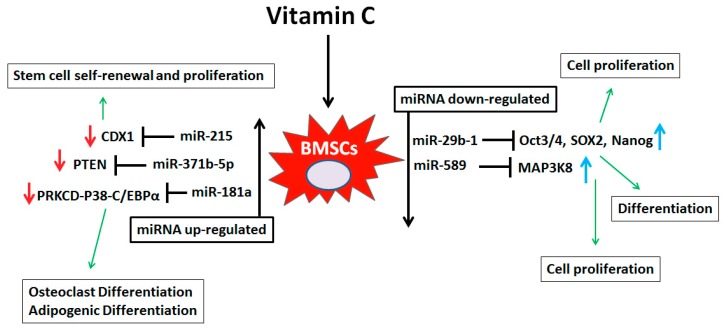
Vitamin C dependent miRNAs mediated signaling in human bone marrow stromal cells. Schematic diagram showing the contribution of key miRNAs in cell proliferation and differentiation of human bone marrow stromal cell.

We correlated our miRNA data with published literature to gain additional insights into the role(s) of these differentially regulated miRNAs in stem cell biology. Scientific literature suggests that miRNAs differentially regulated by vitamin C have an important role in mouse and human stem cell biology. Di Fiore et al (2014) reported that miR-29b-1 regulates cell proliferation, clonogenic growth, and migration ability of osteosarcoma cells, through negative regulation of stemness markers (Oct3/4, Sox2 and Nanog) [56]. Zhang et al. (2016) demonstrated that miR-589-5p inhibits MAP3K8 and suppresses CD90+ cancer stem cells in hepatocellular carcinoma [57]. Both miR-29b-1 and miR-589-5p are down-regulated in vitamin C- treated human BMSCs. There is a possibility that vitamin C treatment down-regulates miR-29b-1 and miR-589-5p and promotes Oct3/4, Sox2, Nanog and SOX2 and MAP3K8 expression respectively in hBMSCs and helps in cell proliferation and differentiation. Moreover, Quan et al. (2017) showed that human alveolar progenitor type II cell (ATIIC)-derived exosomal miR-371b-5p promotes ATIIC-specific stem cells proliferation by targeting PTEN (phosphatase and tensin homolog) [58]. Su et al. (2015) demonstrated that miR-181a inhibits differentiation of HL-60 cells and CD34+ hematopoietic stem/progenitor cells by directly targeting PRKCD-P38-C/EBPα pathway [59] and Jones et al. (2015) reported that miR-215 target caudal-type homeobox 1 (CDX1) and regulates colorectal cancer stem cell differentiation [60]. The above mentioned miRNAs (miR-371b-5p, miR-181a and miR-215) were up-regulated with treatment of vitamin C to hBMSCs. We speculate that these miRNAs target PTEN, PRKCD-P38-C/EBPα and CDX1, respectively, and promote cell proliferation and differentiation of BMSCs. Figure 5 showing possible vitamin C dependent miRNAs mediated signaling in bone marrow stromal cells based on published verified targets.

Our miRNA array data identified a number of novel miRNAs; their functions are not well-understood. In order to determine the potential role(s) of miRNAs involved in hBMSCs exposed to vitamin C, we analyzed the predicted target genes of selected miRNAs. Bioinformatics analysis is an important tool for identifying the predicted role of novel miRNAs. We focused on selected miRNAs: miR-3619-5p, miR-548a-3p, miR-3942-5p, miR-4741, miR-1825, and miR-1208. After bioinformatics analysis, we found that these miRNAs target a number of genes that are important for regulating stem cell differentiation, particularly with regard to lineage commitment in musculoskeletal tissues (See Table 4). MicroRNA gene regulation is a complex process. It is well known that a single miRNA can target a number of genes, and a single gene is targeted by a number of miRNAs. We hypothesized that vitamin C dependent hBMSC cell proliferation and differentiation is partly regulated by these novel miRNAs.

To determine the biological function of miRNAs that are differentially expressed by vitamin C, KEGG pathway annotation and GO analysis were performed to analyze their target gene pools. KEGG annotation revealed that a number of signaling molecules are regulated by vitamin C treatment. Perhaps the most important signaling molecules are cell adhesion molecules (CAMs), fatty acid biosynthesis/metabolism and thyroid hormone signaling pathway molecules. These signaling molecules are regulated by both down-regulated and up-regulated miRNAs. Previously, Pustylnik et al. (2013) demonstrated that vitamin C induces the expression of cell adhesion molecules and stimulates the differentiation of osteoblasts [61]. Furthermore, it has been previously reported that supplementation of vitamin C improves the thyroid hormone level in hypothyroidism patients [62]. Dysregulation of thyroid hormone is involved in various musculoskeletal pathologies such as osteoporosis [63,64]. Our KEGG signaling analysis indicated that vitamin C might regulate miRNAs of thyroid hormone importance and may participate in bone metabolism. Bayerle-Eder et al. (2004) reported that supplementation with vitamin C improves lipid-induced impairment of endothelium-dependent vasodilation [65] and Ginter et al. (1969) demonstrated that chronic vitamin C deficiency affects the fatty acid composition of blood serum, liver triglyceride and cholesterol [66]. Fatty acids and their metabolites are important factors in stem cell proliferation and differentiation [67,68]. Our study indicates that vitamin C-induced miRNAs might have a substantial role in fatty acid-dependent signaling in BMSC cell biology. Moreover, the GO analysis showed that vitamin C is involved in a number of cellular metabolic processes (such as protein biosynthesis, cellular functions, enzyme biosynthesis, cell cycle) and signaling (such as TLR, TRK, and MAPK signaling) (Table 3). These cellular metabolic processes and signaling are vital in BMSC proliferation and differentiation.

## 5. Conclusions

To our knowledge, our study is the first report to identify vitamin C-dependent differential novel miRNA expression in hBMSCs. We also provide new data linking these miRNAs to key signaling pathways involved in BMSC biology. We believe our study is a key step in gaining a greater understanding of how vitamin C contributes to hBMSC biology through the miRNA signaling network. Our study has some limitations. For example, we performed the study only on human bone marrow derived stromal cells, and the vitamin C-dependent miRNA regulation in adipose-derived stem cells could potentially differ somewhat from hBMSCs. Similar studies need to be performed on different stem cells originating from other tissue types. Furthermore, we performed our study only at one-time point (six-day) after treatment with vitamin C. It is important to know both the short and long term effects of vitamin C on BMSCs. Future work by our group will focus on validating the predicted miRNA targets and their role in vitamin C-dependent tissue engineering and musculoskeletal development.

## Figures and Tables

**Table 1:** Selected miRNAs differentially regulated in presence of vitamin C in human bone marrow stromal cells.

MicroRNA ID/Probeset ID	Fold-Change	*p*-Value
hsa-miR-3651_st	−3.76469	0.000718
hsa-miR-4485_st	−3.70276	0.002619
hsa-miR-1275_st	−3.00098	0.022393
hsa-miR-4708-5p_st	−2.94613	0.002456
hsa-miR-3197_st	−2.86102	0.048879
hsa-miR-720_st	−2.81658	0.019125
hsa-miR-210_st	−2.75739	0.001763
hsa-miR-29b-1-star_st	−2.71192	0.032586
hsa-miR-4284_st	−2.49253	0.003259
hsa-miR-4479_st	−2.39325	0.026759
hsa-miR-3175_st	−2.37271	0.013122
hsa-miR-4730_st	−2.36644	0.009171
hsa-miR-23a-star_st	−2.26148	0.001843
hsa-miR-4321_st	−2.25569	0.024075
hp_hsa-mir-3676_st	−2.13436	0.008900
hsa-miR-4787-3p_st	−2.05594	0.003156
hsa-miR-574-5p_st	−2.02613	0.019283
hsa-miR-4492_st	−1.99221	0.011873
hsa-miR-345_st	−1.96585	0.019316
hsa-miR-1270_st	−1.90283	0.047187
hsa-miR-4697-5p_st	−1.83134	0.039445
hsa-miR-4433_st	−1.80613	0.005357
hp_hsa-mir-4477a_st	1.83543	0.0351021
hp_hsa-mir-548ag-2_st	1.83747	0.0217069
hsa-miR-4727-3p_st	1.86033	0.0446828
hsa-miR-335_st	1.86288	0.0415722
hsa-miR-202_st	1.87834	0.00218913
hsa-miR-4436a_st	1.96484	0.00880198
hp_hsa-mir-532_st	1.98369	0.035662
hsa-miR-3942-5p_st	1.98754	0.002823
hsa-miR-3163_st	2.0011	0.019565
hp_hsa-mir-548f-1_st	2.01894	0.026558
hsa-miR-92a-2-star_st	2.02393	0.003107
hsa-miR-548a-3p_st	2.02776	0.003973
hsa-miR-3121-3p_st	2.03509	0.007681
hsa-miR-3201_st	2.07949	0.006952
hsa-miR-4657_st	2.07988	0.027179
hsa-miR-4704-5p_st	2.10426	0.002415
hsa-miR-1825_st	2.1387	0.028925
hsa-miR-550a-star_st	2.16711	0.017221
hsa-miR-1323_st	2.21591	0.006721
hsa-miR-3927_st	2.23141	0.002801
hsa-miR-509-3-5p_st	2.2723	0.003025
hsa-miR-4423-3p_st	2.27939	0.030500
hsa-miR-890_st	2.34751	0.035107
hsa-miR-4773_st	2.41459	0.002370
hsa-miR-371b-5p_st	2.42868	0.038520
hsa-miR-3128_st	2.49419	0.014536
hsa-miR-1272_st	2.59151	0.033417
hsa-miR-4659a-3p_st	2.60253	0.005995
hsa-miR-377_st	2.64791	0.003494
hsa-miR-550b_st	2.68252	0.013600
hsa-miR-20b-star_st	2.76807	0.004101
hsa-miR-3152-3p_st	3.2214	0.002098
hsa-miR-1208_st	5.02485	0.010838
hsa-miR-4529-3p_st	12.0104	0.009329

**Table 2:** Selected KEGG biological pathways potentially affected by (**a**) miRNAs down-regulated; and (**b**) miRNAs up-regulated in the presence of vitamin C in human bone marrow stromal cells.

**(a)**			
**KEGG Pathway**	***p*-Value**	**Number of Genes Involved**	**Number of miRNAs Involved**
Prion diseases	4.07 × 10^−10^	7	7
Morphine addiction	4.80 × 10^−5^	32	17
Amino sugar and nucleotide sugar metabolism	0.002142	16	11
Thyroid hormone signaling pathway	0.002958	37	17
Cell adhesion molecules (CAMs)	0.006179	43	16
Endocytosis	0.009192	63	18
Oxytocin signaling pathway	0.011941	53	21
Melanogenesis	0.013774	35	17
MAPK signaling pathway	0.013774	77	22
GABAergic synapse	0.02274	32	18
Vasopressin-regulated water reabsorption	0.035605	16	11
Adrenergic signaling in cardiomyocytes	0.035605	48	22
Glutamatergic synapse	0.03746	34	13
Biosynthesis of unsaturated fatty acids	0.040121	7	5
Circadian entrainment	0.046058	37	16
**(b)**			
**KEGG Pathway**	***p*-Value**	**Number of Genes Involved**	**Number of miRNAs Involved**
Mucin type O-Glycan biosynthesis	1.69 × 10^−11^	11	7
Fatty acid biosynthesis	1.86 × 10^−8^	1	1
Glycosphingolipid biosynthesis-lacto and neolacto series	6.27 × 10^−5^	9	8
Fatty acid metabolism	0.000184	7	6
Adherens junction	0.000623	25	18
Biotin metabolism	0.010804	1	1
Arrhythmogenic right ventricular cardiomyopathy	0.014001	21	18
Caffeine metabolism	0.027365	2	2
Thyroid hormone signaling pathway	0.027365	32	21

**Table 3:** Selected (firsts 50) gene ontology (GO) biological pathways potentially affected by (**a**) miRNAs down-regulated; and (**b**) miRNAs up-regulated in the presence of vitamin C in human bone marrow stromal cells.

**(a)**			
**GO Category**	***p*-Value**	**Genes**	**miRNAs**
organelle	1.15 × 10^−87^	2073	24
ion binding	7.43 × 10^−46^	1270	25
cellular nitrogen compound metabolic process	7.82 × 10^−45^	1006	24
biosynthetic process	1.56 × 10^−32^	856	24
small molecule metabolic process	1.56 × 10^−17^	489	24
cellular protein modification process	3.92 × 10^−15^	475	24
neurotrophin TRK receptor signaling pathway	2.05 × 10^−14^	70	18
synaptic transmission	2.05 × 10^−14^	121	21
nucleic acid binding transcription factor activity	1.32 × 10^−13^	231	24
cellular protein metabolic process	2.55 × 10^−11^	106	20
cell-cell signaling	1.27 × 10^−10^	162	24
catabolic process	1.50 × 10^−10^	390	24
cellular component assembly	2.41 × 10^−9^	272	23
molecular_function	2.43 × 10^−9^	3342	25
gene expression	2.95 × 10^−9^	116	22
symbiosis, encompassing mutualism through parasitism	4.66 × 10^−9^	111	21
post-translational protein modification	4.93 × 10^−9^	46	17
cellular_component	5.00 × 10^−9^	3383	25
blood coagulation	1.28 × 10^−8^	101	20
viral process	2.94 × 10^−8^	97	21
protein binding transcription factor activity	7.72 × 10^−8^	110	22
macromolecular complex assembly	1.45 × 10^−7^	185	23
enzyme binding	1.74 × 10^−7^	261	23
Fc-epsilon receptor signaling pathway	5.08 × 10^−7^	38	15
response to stress	1.46 × 10^−6^	437	25
toll-like receptor TLR1:TLR2 signaling pathway	2.79 × 10^−6^	21	10
toll-like receptor TLR6:TLR2 signaling pathway	2.79 × 10^−6^	21	10
toll-like receptor 10 signaling pathway	3.62 × 10^−6^	20	10
membrane organization	5.32 × 10^−6^	121	23
energy reserve metabolic process	8.12 × 10^−6^	30	13
TRIF-dependent toll-like receptor signaling pathway	1.46 × 10^−5^	21	10
protein complex assembly	1.95 × 10^−5^	158	23
MyD88-independent toll-like receptor signaling pathway	2.89 × 10^−5^	22	11
protein complex	3.71 × 10^−5^	708	24
toll-like receptor 5 signaling pathway	3.75 × 10^−5^	20	10
toll-like receptor 9 signaling pathway	4.84 × 10^−5^	21	10
Golgi lumen	5.79 × 10^−5^	26	11
O-glycan processing	7.15 × 10^−5^	18	12
immune system process	0.000127	309	25
mitotic cell cycle	0.00013	74	19
cytoskeletal protein binding	0.000137	155	22
generation of precursor metabolites and energy	0.000171	74	18
regulation of rhodopsin mediated signaling pathway	0.000201	12	10
toll-like receptor 4 signaling pathway	0.000201	26	11
inositol phosphate metabolic process	0.000219	17	9
nucleobase-containing compound catabolic process	0.000385	168	24
toll-like receptor 2 signaling pathway	0.000449	21	10
platelet degranulation	0.000563	20	15
glycosaminoglycan metabolic process	0.000573	26	13
platelet activation	0.000643	44	18
**(b)**			
**GO Category**	***p*-Value**	**Genes**	**miRNAs**
organelle	1.67 × 10^−95^	2091	47
ion binding	3.27 × 10^−55^	1303	47
cellular nitrogen compound metabolic process	7.65 × 10^−47^	1010	46
biosynthetic process	1.19 × 10^−35^	866	46
gene expression	7.04 × 10^−23^	151	38
cellular protein modification process	1.18 × 10^−22^	507	46
small molecule metabolic process	7.20 × 10^−18^	488	45
nucleic acid binding transcription factor activity	1.08 × 10^−12^	227	43
molecular_function	2.35 × 10^−11^	3343	48
cellular_component	8.85 × 10^−10^	3375	48
cellular protein metabolic process	1.27 × 10^−9^	101	35
enzyme binding	1.33 × 10^−9^	272	43
protein binding transcription factor activity	5.19 × 10^−9^	114	37
cellular component assembly	9.08 × 10^−9^	268	42
viral process	1.39 × 10^−8^	98	31
blood coagulation	2.54 × 10^−8^	100	35
symbiosis, encompassing mutualism through parasitism	6.42 × 10^−8^	107	32
neurotrophin TRK receptor signaling pathway	9.66 × 10^−8^	56	28
catabolic process	9.95 × 10^−8^	371	44
cell death	3.44 × 10^−7^	195	40
protein complex	5.91 × 10^−7^	724	46
enzyme regulator activity	7.70 × 10^−7^	179	36
platelet activation	9.11 × 10^−7^	52	25
membrane organization	2.77 × 10^−6^	122	36
post-translational protein modification	3.62 × 10^−6^	40	23
Fc-gamma receptor signaling pathway involved in phagocytosis	4.22 × 10^−6^	22	13
cytoskeletal protein binding	1.60 × 10^−5^	160	37
nucleoplasm	1.79 × 10^−5^	233	43
cytosol	2.00 × 10^−5^	523	43
cell junction organization	2.35 × 10^−5^	42	24
nucleobase-containing compound catabolic process	3.39 × 10^−5^	174	41
transmembrane transporter activity	3.63 × 10^−5^	219	38
macromolecular complex assembly	3.63 × 10^−5^	172	39
toll-like receptor TLR1:TLR2 signaling pathway	5.04 × 10^−5^	19	15
toll-like receptor TLR6:TLR2 signaling pathway	5.04 × 10^−5^	19	15
TRIF-dependent toll-like receptor signaling pathway	5.32 × 10^−5^	20	16
toll-like receptor 10 signaling pathway	6.86 × 10^−5^	18	15
Fc-epsilon receptor signaling pathway	8.73 × 10^−5^	33	19
cellular component disassembly involved in execution phase of apoptosis	0.000104	15	11
vitamin metabolic process	0.000237	21	19
MyD88-independent toll-like receptor signaling pathway	0.000351	20	16
homeostatic process	0.000351	168	41
mitotic cell cycle	0.000368	72	34
protein complex assembly	0.000392	150	37
water-soluble vitamin metabolic process	0.000403	19	17
toll-like receptor 5 signaling pathway	0.000491	18	15
energy reserve metabolic process	0.000538	26	18
glycerophospholipid biosynthetic process	0.000692	23	14
activation of signaling protein activity involved in unfolded protein response	0.000692	18	16
transcription initiation from RNA polymerase II promoter	0.000793	52	25
toll-like receptor 9 signaling pathway	0.001896	18	15

**Table 4:** Predicated targets of differentially regulated miRNAs of stem cell biology.

miRNA	No. of Targets		Stem Cell Related Genes
	MiRDB Scan	Target Scan	Common Targets
hsa-miR-3619-5p	788	5948	PPARGC1B, RUNX3, DLX3, TGFBRAP1, TNFAIP1, TRAF1, TRAF3, TRAF5, TNFAIP8L1, MMP24, SOX8, QSOX2, WNT3
hsa-miR-548a-3p	947	6717	BMPR2, TGFBR3, TGFBR1, TAB2, TWISTNB, CDC42BPB, IL6R, TRAF6, SMAD4, SMAD1, SMAD5, MMP2, WNT3
hsa-miR-3942-5p	437	3758	DLX1, BMPR2, BMP2K, TGFB2, IL6R, TNFRSF11A, TRAF3, SMAD1
hsa-miR-4741	253	4549	TAB2, SMURF1, SNIP1
hsa-miR-1825	253	4160	PPARD, RUNX2, TGFBRAP1, TGFBR1, CD40, SOX6
hsa-miR-1208	304	4651	TGFB2, TWISTNB, SMURF1, MMP16, LEPROT

## Supplementary Materials

The following are available online at http://www.mdpi.com/2072-6643/10/2/186/s1, Figure S1: Real-time PCR showing no significant changes in miRNA expression in low dose (25 µM) vitamin C treated samples compared to control (a) miR-4708, (b) miR-29b, (c) miR-3152, and (d) miR-3942 (*n* = 4), Figure S2: Wordle-based clouds for combine differentially (up and down-regulated) regulated miRNAs of vitamin C treated samples (a) KEGG and (b) GO analysis. Word clouds demonstrating the font size depending on relative word frequencies in KEGG and GO analysis, Table S1: Nucleotide sequences of human primers used for RT-PCR.

## References

[1] Jones D.L., Wagers A.J. (2008). No place like home: Anatomy and function of the stem cell niche. Nat. Rev. Mol. Cell Biol..

[2] Li W., Wei S., Liu C., Song M., Wu H., Yang Y. (2016). Regulation of the osteogenic and adipogenic differentiation of bone marrow-derived stromal cells by extracellular uridine triphosphate: The role of P2Y2 receptor and ERK1/2 signaling. Int. J. Mol. Med..

[3] Xue J.X., Gong Y.Y., Zhou G.D., Liu W., Cao Y., Zhang W.J. (2012). Chondrogenic differentiation of bone marrow-derived mesenchymal stem cells induced by acellular cartilage sheets. Biomaterials.

[4] Rahman F., Bordignon B., Culerrier R., Peiretti F., Spicuglia S., Djabali M., Landrier J.F., Fontes M. (2017). Ascorbic acid drives the differentiation of mesoderm-derived embryonic stem cells. Involvement of p38 MAPK/CREB and SVCT2 transporter. Mol. Nutr. Food Res..

[5] Kilberg M.S., Terada N., Shan J. (2016). Influence of Amino Acid Metabolism on Embryonic Stem Cell Function and Differentiation. Adv. Nutr..

[6] Ochocki J.D., Simon M.C. (2013). Nutrient-sensing pathways and metabolic regulation in stem cells. J. Cell Biol..

[7] Khillan J.S. (2014). Vitamin A/retinol and maintenance of pluripotency of stem cells. Nutrients.

[8] Sakayori N., Kimura R., Osumi N. (2013). Impact of lipid nutrition on neural stem/progenitor cells. Stem Cells Int..

[9] Sangani R., Periyasamy-Thandavan S., Pathania R., Ahmad S., Kutiyanawalla A., Kolhe R., Bhattacharyya M.H., Chutkan N., Hunter M., Hill W.D. (2015). The crucial role of vitamin C and its transporter (SVCT2) in bone marrow stromal cell autophagy and apoptosis. Stem Cell Res..

[10] Bae S.H., Ryu H., Rhee K.J., Oh J.E., Baik S.K., Shim K.Y., Kong J.H., Hyun S.Y., Pack H.S., Im C. (2015). l-Ascorbic acid 2-phosphate and fibroblast growth factor-2 treatment maintains differentiation potential in bone marrow-derived mesenchymal stem cells through expression of hepatocyte growth factor. Growth Factors.

[11] Aghajanian P., Hall S., Wongworawat M.D., Mohan S. (2015). The Roles and Mechanisms of Actions of Vitamin C in Bone: New Developments. J. Bone Miner. Res..

[12] Fain O. (2005). Musculoskeletal manifestations of scurvy. Joint Bone Spine.

[13] Fulzele S., Chothe P., Sangani R., Chutkan N., Hamrick M., Bhattacharyya M., Prasad P.D., Zakhary I., Bowser M., Isales C. (2013). Sodium-dependent vitamin C transporter SVCT2, expression and function in bone marrow stromal cells and in osteogenesis. Stem Cell Res..

[14] Choi K.M., Seo Y.K., Yoon H.H., Song K.Y., Kwon S.Y., Lee H.S., Park J.K. (2008). Effect of ascorbic acid on bone marrow-derived mesenchymal stem cell proliferation and differentiation. J. Biosci. Bioeng..

[15] Potdar P.D., D’Souza S.B. (2010). Ascorbic acid induces in vitro proliferation of human subcutaneous adipose tissue derived mesenchymal stem cells with upregulation of embryonic stem cell pluripotency markers Oct4 and SOX2. Hum. Cell.

[16] Naidu K.A. (2003). Vitamin C in human health and disease is still a mystery? An overview. Nutr. J..

[17] Drouin G., Godin J.R., Pagé B. (2011). The genetics of vitamin C loss in vertebrates. Curr. Genomics.

[18] Nishikimi M., Yagi K. (1991). Molecular basis for the deficiency in humans of gulonolactone oxidase, a key enzyme for ascorbic acid biosynthesis. Am. J. Clin. Nutr..

[19] Chung T.L., Brena R.M., Kolle G., Grimmond S.M., Berman B.P., Laird P.W., Pera M.F., Wolvetang E.J. (2010). Vitamin C promotes widespread yet specific DNA demethylation of the epigenome in human embryonic stem cells. Stem Cells.

[20] Blaschke K., Ebata K.T., Karimi M.M., Zepeda-Martínez J.A., Goyal P., Mahapatra S., Tam A., Laird D.J., Hirst M., Rao A. (2013). Vitamin C induces Tet-dependent DNA demethylation and a blastocyst-like state in ES cells. Nature.

[21] Poal-Manresa J., Little K., Trueta J. (1970). Some observations on the effects of vitamin C deficiency on bone. Br. J. Exp. Pathol..

[22] Kipp D.E., McElvain M., Kimmel D.B., Akhter M.P., Robinson R.G., Lukert B.P. (1996). Scurvy results in decreased collagen synthesis and bone density in the guinea pig animal model. Bone.

[23] Chen J., Guo L., Zhang L., Wu H., Yang J., Liu H., Wang X., Hu X., Gu T., Zhou Z. (2013). Vitamin C modulates TET1 function during somatic cell reprogramming. Nat. Genet..

[24] Ivanyuk D., Budash G., Zheng Y., Gaspar J.A., Chaudhari U., Fatima A., Bahmanpour S., Grin V.K., Popandopulo A.G., Sachinidis A. (2015). Ascorbic Acid-Induced Cardiac Differentiation of Murine Pluripotent Stem Cells: Transcriptional Profiling and Effect of a Small Molecule Synergist of Wnt/β-Catenin Signaling Pathway. Cell. Physiol. Biochem..

[25] Weinhold B. (2006). Epigenetics: The science of change. Environ. Health Perspect..

[26] Fu G., Ren A., Qiu Y., Zhang Y. (2016). Epigenetic Regulation of Osteogenic Differentiation of Mesenchymal Stem Cells. Curr. Stem Cell Res. Ther..

[27] Yannarelli G., Pacienza N., Cuniberti L., Medin J., Davies J., Keating A. (2013). Brief report: The potential role of epigenetics on multipotent cell differentiation capacity of mesenchymal stromal cells. Stem Cells.

[28] Challen G.A., Sun D., Jeong M., Luo M., Jelinek J., Berg J.S., Bock C., Vasanthakumar A., Gu H., Xi Y. (2011). Dnmt3a is essential for hematopoietic stem cell differentiation. Nat. Genet..

[29] Arnsdorf E.J., Tummala P., Castillo A.B., Zhang F., Jacobs C.R. (2010). The epigenetic mechanism of mechanically induced osteogenic differentiation. J. Biomech..

[30] Alexanian A.R. (2007). Epigenetic modifiers promote efficient generation of neural-like cells from bone marrow-derived mesenchymal cells grown in neural environment. J. Cell. Biochem..

[31] Sangani R., Periyasamy-Thandavan S., Kolhe R., Bhattacharyya M.H., Chutkan N., Hunter M., Isales C., Hamrick M., Hill W.D., Fulzele S. (2015). MicroRNAs-141 and 200a regulate the SVCT2 transporter in bone marrow stromal cells. Mol. Cell. Endocrinol..

[32] Ebata K.T., Mesh K., Liu S., Bilenky M., Fekete A., Acker M.G., Hirst M., Garcia B.A., Ramalho-Santos M. (2017). Vitamin C induces specific demethylation of H3K9me2 in mouse embryonic stem cells via Kdm3a/b. Epigenet. Chromatin.

[33] Abbey D., Seshagiri P.B. (2017). Ascorbic acid-mediated enhanced cardiomyocytes differentiation of mouse ES-cells involves interplay of DNA methylation and multiple-signals. Differentiation.

[34] Eid W., Abdel-Rehim W. (2016). Vitamin C promotes pluripotency of human induced pluripotent stem cells via the histone demethylase JARID1A. Biol. Chem..

[35] Camarena V., Wang G. (2016). The epigenetic role of vitamin C in health and disease. Cell. Mol. Life Sci..

[36] Young J.I., Züchner S., Wang G. (2015). Regulation of the Epigenome by Vitamin C. Annu. Rev. Nutr..

[37] Yin R., Mao S.Q., Zhao B., Chong Z., Yang Y., Zhao C., Zhang D., Huang H., Gao J., Li Z. (2013). Ascorbic acid enhances Tet-mediated 5-methylcytosine oxidation and promotes DNA demethylation in mammals. J. Am. Chem. Soc..

[38] Nicolas F.E., Lopez-Martinez A.F. (2010). MicroRNAs in human diseases. Recent Pat. DNA Gene Seq..

[39] He L., Hannon G.J. (2004). MicroRNAs: Small RNAs with a big role in gene regulation. Nat. Rev. Genet..

[40] Cruz-Santos M.C., Aragón-Raygoza A., Espinal-Centeno A., Arteaga-Vázquez M., Cruz-Hernández A., Bako L., Cruz-Ramírez A. (2016). The Role of microRNAs in Animal Cell Reprogramming. Stem Cells Dev..

[41] Mathieu J., Ruohola-Baker H. (2013). Regulation of stem cell populations by microRNAs. Adv. Exp. Med. Biol..

[42] Luo W., Nie Q., Zhang X. (2013). MicroRNAs involved in skeletal muscle differentiation. J. Genet. Genomics.

[43] Yi R., Fuchs E. (2011). MicroRNAs and their roles in mammalian stem cells. J. Cell Sci..

[44] Calabrese G., Giuffrida R., Lo Furno D., Parrinello N.L., Forte S., Gulino R., Colarossi C., Schinocca L.R., Giuffrida R., Cardile V. (2015). Potential Effect of CD271 on Human Mesenchymal Stromal Cell Proliferation and Differentiation. Int. J. Mol. Sci..

[45] Cuthbert R.J., Giannoudis P.V., Wang X.N., Nicholson L., Pawson D., Lubenko A., Tan H.B., Dickinson A., McGonagle D., Jones E. (2015). Examining the feasibility of clinical grade CD271 + enrichment of mesenchymal stromal cells for bone regeneration. PLoS ONE.

[46] Kolhe R., Hunter M., Liu S., Jadeja R.N., Pundkar C., Mondal A.K., Mendhe B., Drewry M., Rojiani M.V., Liu Y. (2017). Gender-specific differential expression of exosomal miRNA in synovial fluid of patients with osteoarthritis. Sci. Rep..

[47] Tuan R.S., Boland G., Tuli R. (2003). Adult mesenchymal stem cells and cell-based tissue engineering. Arthritis Res. Ther..

[48] Tang X.L., Xu M.J., Li Z.H., Pan Q., Fu J.H. (2013). Effects of vitamin E on expressions of eight microRNAs in the liver of Nile tilapia (Oreochromis niloticus). Fish Shellfish Immunol..

[49] Gaedicke S., Zhang X., Schmelzer C., Lou Y., Doering F., Frank J., Rimbach G. (2008). Vitamin E dependent microRNA regulation in rat liver. FEBS Lett..

[50] Merrigan S.L., Kennedy B.N. (2017). Vitamin D receptor agonists regulate ocular developmental angiogenesis and modulate expression of dre-miR-21 and VEGF. Br. J. Pharmacol..

[51] Arboleda J.F., Urcuqui-Inchima S. (2016). Vitamin D-Regulated MicroRNAs: Are They Protective Factors against Dengue Virus Infection?. Adv. Virol..

[52] Drummond M.J., Glynn E.L., Fry C.S., Dhanani S., Volpi E., Rasmussen B.B. (2009). Essential amino acids increase microRNA-499, -208b, and -23a and downregulate myostatin and myocyte enhancer factor 2C mRNA expression in human skeletal muscle. J. Nutr..

[53] Kim S.M., Lim S.M., Yoo J.A., Woo M.J., Cho K.H. (2015). Consumption of high-dose vitamin C (1250 mg per day) enhances functional and structural properties of serum lipoprotein to improve anti-oxidant, anti-atherosclerotic, and anti-aging effects via regulation of anti-inflammatory microRNA. Food Funct..

[54] Gao Y., Han Z., Li Q., Wu Y., Shi X., Ai Z., Du J., Li W., Guo Z., Zhang Y. (2015). Vitamin C induces a pluripotent state in mouse embryonic stem cells by modulating microRNA expression. FEBS J..

[55] Kim Y.J., Ku S.Y., Rosenwaks Z., Liu H.C., Chi S.W., Kang J.S., Lee W.J., Jung K.C., Kim S.H., Choi Y.M. (2010). MicroRNA expression profiles are altered by gonadotropins and vitamin C status during in vitro follicular growth. Reprod. Sci..

[56] Di Fiore R., Drago-Ferrante R., Pentimalli F., Di Marzo D., Forte I.M., D’Anneo A., Carlisi D., De Blasio A., Giuliano M., Tesoriere G. (2014). MicroRNA-29b-1 impairs in vitro cell proliferation, self-renewal and chemoresistance of human osteosarcoma 3AB-OS cancer stem cells. Int. J. Oncol..

[57] Zhang X., Jiang P., Shuai L., Chen K., Li Z., Zhang Y., Jiang Y., Li X. (2016). miR-589-5p inhibits MAP3K8 and suppresses CD90(+) cancer stem cells in hepatocellular carcinoma. J. Exp. Clin. Cancer Res..

[58] Quan Y., Wang Z., Gong L., Peng X., Richard M.A., Zhang J., Fornage M., Alcorn J.L., Wang D. (2017). Exosome miR-371b-5p promotes proliferation of lung alveolar progenitor type II cells by using PTEN to orchestrate the PI3K/Akt signaling. Stem Cell Res. Ther..

[59] Su R., Lin H.S., Zhang X.H., Yin X.L., Ning H.M., Liu B., Zhai P.F., Gong J.N., Shen C., Song L. (2015). MiR-181 family: Regulators of myeloid differentiation and acute myeloid leukemia as well as potential therapeutic targets. Oncogene.

[60] Jones M.F., Hara T., Francis P., Li X.L., Bilke S., Zhu Y., Pineda M., Subramanian M., Bodmer W.F., Lal A. (2015). The CDX1-microRNA-215 axis regulates colorectal cancer stem cell differentiation. Proc. Natl. Acad. Sci. USA.

[61] Pustylnik S., Fiorino C., Nabavi N., Zappitelli T., da Silva R., Aubin J.E., Harrison R.E. (2013). EB1 levels are elevated in ascorbic Acid (AA)-stimulated osteoblasts and mediate cell-cell adhesion-induced osteoblast differentiation. J. Biol. Chem..

[62] Jubiz W., Ramirez M. (2014). Effect of vitamin C on the absorption of levothyroxine in patients with hypothyroidism and gastritis. J. Clin. Endocrinol. Metab..

[63] Williams G.R., Bassett J.H.D. (2017). Thyroid diseases and bone health. J. Endocrinol. Investig..

[64] Williams G.R. (2013). Thyroid hormone actions in cartilage and bone. Eur. Thyroid. J..

[65] Bayerle-Eder M., Pleiner J., Mittermayer F., Schaller G., Roden M., Waldhäusl W., Bieglmayer C., Wolzt M. (2004). Effect of systemic vitamin C on free fatty acid-induced lipid peroxidation. Diabetes Metab..

[66] Ginter E., Ondreicka R., Bobek P., Simko V. (1969). The influence of chronic vitamin C deficiency on fatty acid composition of blood serum, liver triglycerides and cholesterol esters in guinea pigs. J. Nutr..

[67] Rashid M.A., Haque M., Akbar M. (2016). Role of Polyunsaturated Fatty Acids and Their Metabolites on Stem Cell Proliferation and Differentiation. Adv. Neurobiol..

[68] Das U.N. (2011). Influence of polyunsaturated fatty acids and their metabolites on stem cell biology. Nutrition.

