# Supplementary material for: Modulation of miRNAs by Vitamin C in Human Bone Marrow Stromal Cells
Source: Nutrients. 2018 Feb 8;10(2):186. doi: 10.3390/nu10020186 (PMC5852762; doi:10.3390/nu10020186)
Supplement: Supplementary file 1 [file nutrients-10-00186-s001.pdf]

Supplementary figures

Figure S1. Real-time PCR showing no significant changes in miRNA expression in low dose (25µM) vitamin C treated samples compared to control (a) miR-4708 , b) miR-29b, c) miR-3152, and d) miR-3942 (n=4).

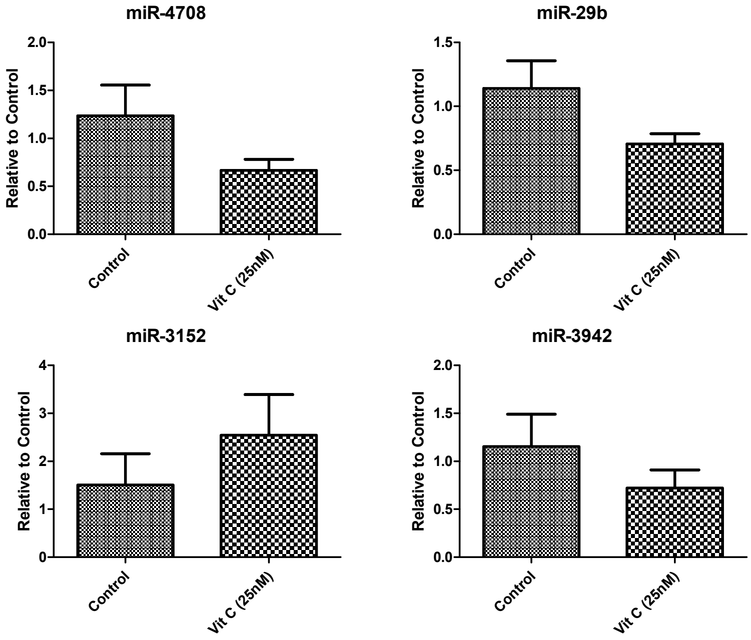

Figure S2. Wordle-based clouds for combine differentially (up and down-regulated) regulated miRNAs of vitamin C treated samples (a) KEGG and (b) GO fanalysis. Word clouds demonstrating the font size depending on relative word frequencies in KEGG and GO analysis.

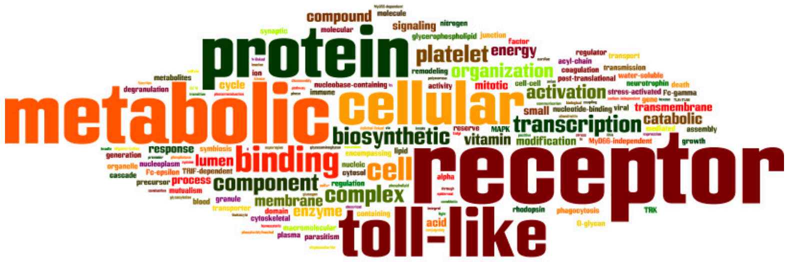

Table.S1 Nucleotide sequences of human primers used for RT-PCR

Gene	Primer	Reference/Accession Number
β-actin	ACA TGT ATG AAG GCT TTT GGT CTC<br>GTG TGC ACT TTT ATT CAA CTG GTC	NM_001101.3
BMP-2	ATG TTA GGA TAA GCA GGT CTT TGC<br>GAC CTT TTT CTC TTT TGT GGA GAG	NM_001200.2
RUNX-2	GTA CCA GAT GGG ACT GTG GTT ACT<br>CTC AGA TCG TTG AAC CTT GCT ACT	NM_001015051.3
BMP-7	CCT ACC CCT ACA AGG CCG TCT TC<br>TGC TCC CCG TGG ACC GGA TGC TG	[1]
COL-II	CTG CAA AAT AAA ATC TCG GTG TTC T<br>GGG CAT TTG ACT CAC ACC AGT	[2]
OSX	CTG CCT TGG GTT TAT AGA CAT CTT<br>ATC TGA CTT TGC TCC CCT TAA TC	XM_011537900

Reference

1. Yoo, H.J.; Yoon, S.S.; Park, S.Y.; Lee, E.Y.; Lee, E.B.; Kim, J.H.; Song, Y.W. Gene expression profile during chondrogenesis in human bone marrow derived mesenchymal stem cells using a cDNA microarray. *J. Korean Med. Sci.* **2011**, *26*, 851–858.
2. Khan, W.S.; Tew, S.R.; Adesida, A.B.; Hardingham, T.E. Human infrapatellar fat pad-derived stem cells express the pericyte marker 3G5 and show enhanced chondrogenesis after expansion in fibroblast growth factor-2. *Arthritis Res. Ther.* **2008**, *10*, R74.
